# Comparison between absorbable pins and mini-screw fixations for the treatment of radial head fractures Mason type II-III

**DOI:** 10.1186/s12891-018-2014-x

**Published:** 2018-03-27

**Authors:** Luigi Tarallo, Raffaele Mugnai, Martina Rocchi, Francesco Capra, Fabio Catani

**Affiliations:** 1 Department of Orthopaedics and Traumatology, University of Modena and Reggio Emilia, Policlinico di Modena, Modena, Italy; 2 Private Practitioner, Rimini, Italy

**Keywords:** Radial head fracture, Mason, Pin, Mini-screws, Complications, Fixation

## Abstract

**Background:**

The treatment of comminuted radial head fractures can include prosthetic replacement or open reduction and internal fixation. The purpose of this study is to evaluate the results of two different internal fixation systems for Mason type II-III radial head fractures.

**Methods:**

Between 2005 and 2015, 82 patients were treated using pins and 65 patients by mini-screws. The follow-up protocol included: a clinical evaluation 15 days after surgery, and clinical and radiographic evaluations performed at 30 and 60 day intervals, unless any complications were reported by the patient. Over a period of at least 12-months of follow-up, patients were checked and interviewed. Clinical examinations included elbow range of motion (ROM), arm, shoulder and hand Disabilities, (DASH), and the Mayo Elbow Performance Score (MEPS).

**Results:**

Sixty-one subjects who had been treated with mini-screws were clinically reviewed at a mean 47.3 ± 35.8 month of follow-up; all patients who had been treated using absorbable pins were evaluated at a mean 82.5 ± 20.6 month of follow-up. No significant statistically differences were observed between the two groups in the mean ROM, DASH, and MEPS scores. Residual pain was reported in 15.8%of the patients treated by pins and 9.2% patients treated by mini-screws. Secondary displacement of fracture fragments was observed in 8.5% patients treated by pins and 1.6% using mini-screws.

**Conclusions:**

Both absorbable pins and mini-screws provided adequate strength and rigidity, allowing good clinical and functional scores at a mid-term follow-up. However, a higher rate of secondary displacement of the fracture fragments was reported among subjects who had been treated using absorbable pins.

## Background

The treatment of radial head fractures, for a long time was treated with excision of the fracture fragments. This kind of treatment could lead to instability, proximal migration of the radius, joint stiffness and a mismatch at the radio-ulnar joint [1, 2].

Recently the radial head be fixed and the reduction can be maintained thanks to the new techniques obtained in open-reduction and internal fixation (ORIF) [3].

Recent studies comparing ORIF to alternative treatments (resection or replacement) have shown that fixation provides very favourable results in terms of function in fractures characterized by only a few fragments, while very comminute patterns appear to be best managed with arthroplasty or resection [4–7].

The most commonly employed systems for classifying radial head fractures is the Mason classification [8], originally introduced in the 1950s, and recently modified by Hotchkiss [5]. Type I includes non-displaced or minimally displaced (< 2 mm) fracture of the radial head or neck, or marginal lip fracture. Type II partial articular fractures with displacement > 2 mm. Type III severely comminuted fractures involving the entire radial head. Type IV fractures associated with elbow displacement [9].

The aim of this study is to evaluate the results of two different kinds of internal fixations (absorbable pins and mini-screws) in patients with Mason type II and III radial head fracture.

## Methods

### Patients

This retrospective research was approved by the local institutional ethics committee and was carried out following the guidelines of the ethical standards of the 1964 Declaration of Helsinki as revised in 2000. Between 2005 and 2015, 147 patients (age > 18 years) with radial head fracture Mason type II-III were treated using absorbable pins or mini-screws at the authors’ institute, which is a referred trauma and hand surgery center. The only criteria of exclusion to our study is age < 18 years old.

### Surgical technique

**Fig. 1:**
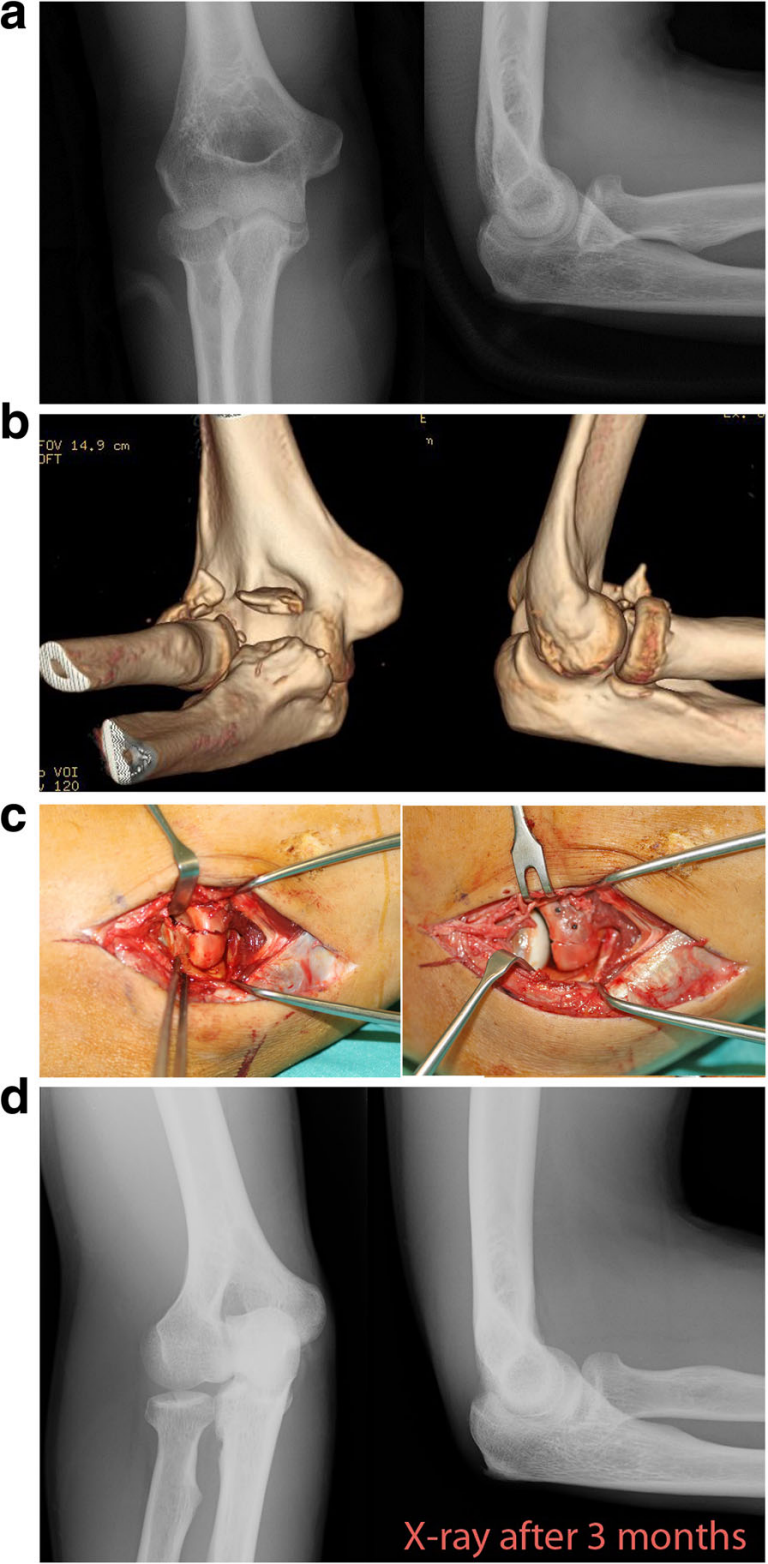
**a** X-rays showing a Mason type II fracture. **b** 3D-computed tomography evaluation confirming a partial articular fracture with high displacement of the fractured fragments. **c** Intra-operative view showing the reduction and fixation using 2 absorbable pins. **d** X-rays performed after 3 months showing healing of the fracture without signs of secondary displacement of the fragments

**Fig. 2:**
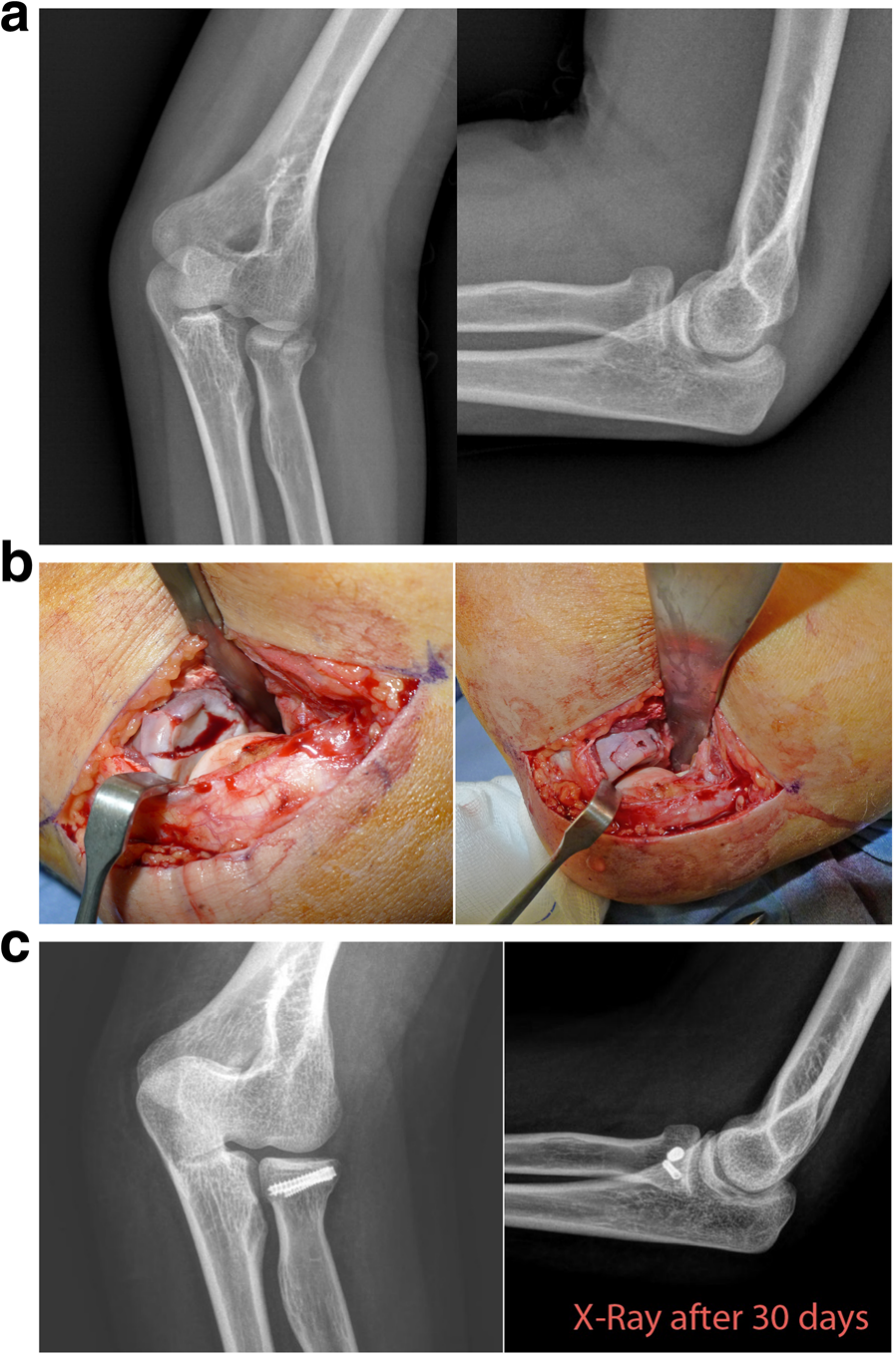
**a** X-rays showing a Mason type II fracture. **b** Intra-operative view showing the reduction and fixation using 2 mini-screws. **c** X-rays performed after 30-days with signs of fracture healing and reduction maintenance

With the patient placed in the supine position, Kocher’s lateral technique was used to expose the radial head between the anconeus and extensor carpi ulnaris. Once reduced, the fracture fragments were fixed with absorbable pins (Fig. 1) or mini-screws (Fig. 2). The choice of the internal fixation system for osteosynthesis was determined by time criterion and independent from subjective characterization: before 2010 only pins were used; later mini-screws became the choice of fixation. In particular the 2 fixation systems employed were:

- Resorbable pins diameter 2.0 mm Orthosorb LS (Lactosorb), composed of 82% Poly-L-lactic acid and 18% Poly-glycolic acid (Zimmer, Biomet).
- Headless compression screws Acutrak mini diameter 3.2 mm (Acumed, Hillsboro, OR).

All procedures were performed by or under the direct supervision of one senior surgeon.

When present, concomitant LCL injuries were treated primarily with bone anchor or trans-osseous bone tunnel. More complex lesions were also repaired at the same time to achieve a complete articular stabilization. A long-arm splint was applied with the elbow in 90° of flexion.

### Outcomes

The post-operative protocol included a clinical evaluation 15 days after surgery, during which the stitches were removed and in the mini-screws group the long-arm splint was substituted with an articulated elbow brace to allow early mobilization, whereas patients treated by pins were maintained immobilized for about one month, according to the Hirvensalo E. and Pelto K. technique [17, 18]. A clinical and radiographic evaluation was also performed after 30 days and 60 days, unless any complications occurred .

Each patient was finally re-evaluated at a minimum of 12-month follow-up (respectively at a mean 82.5 ± 20.6 month for patients treated using pins, and 47.3 ± 35.8 month using mini-screws) by one of the authors, including elbow Range of Motion (ROM) and the following questionnaires: Arm, Shoulder and Hand Disabilities, (DASH) score, and the Mayo Elbow Performance Score (MEPS) [10, 11].

Any possible adverse reactions including residual pain, symptoms of instability of the elbow, early or late occurrence of clinically evident seroma formation, osteolytic changes of the radial head, non-unions, infection or loss of fracture reduction were evaluated, and recorded. In particular, the secondary possible displacement using the hospital X-ray program was measured by the author. In the case of displacement, three different points of the fragment had been selected to take the measurement from the articular surface and the mean value was also taken in consideration. The assessments of radiographic outcomes and clinical outcomes were not blinded.

### Statistical analysis

A summary and statistical analysis is shown in Table 2 (continuous data were expressed as mean ± standard deviation).

The T-test method was followed to define the two-sided probability of statistical significance in Age comparison due to the F-test more than 0.05 (the variances of the two samples can be assumed to be equal). The other analysis has not presented parametric distribution so the Mann-Whitney test for independent samples was carried out. Post-hoc power calculator was introduced to evaluate the statistical strength of the trial. Statistical analysis was monitored using MedCalc for Windows, version 12.2.1 (MedCalc Software, Mariakerke, Belgium).

## Results

The study of population was composed of 74 men and 73 women. The left elbow was affected in 75 patients (7 dominant), whereas the right elbow in 72 patients (69 dominant). The mean age of the subjects treated by mini-screws and pins was 47.2 ± 15.4 and 45.4 ± 13.1 years, respectively. Absorbable pins were used in 82 patients, mini-screws in 65 subjects. Pin population was composed of 61 Mason II and 21 Mason III fractures, while mini-screw group included 47 Mason II and 18 Mason III fractures. A complex injury pattern was present in 20 patients: a concomitant olecranon fracture in 5 cases, coronoid fracture in 10 cases, lateral column distal humerus fracture in 4 patients, and 1 patients had a complex arm injury (concomitant fractures of the scaphoid and scapho-lunate dissociation). The lateral collateral ligament (LCL) was treated in 12 patients. Table 1 lists the concomitant injuries of both fixation methods, mini-screws and pins, and their statistical comparison showed that there were no significant differences between the two study groups.

**Table 1:** Associated injuries

Concomitant Injury	PIN (*n* = 82) N (%)	Mini-screws (*n* = 65) N (%)	*p*-value
Olecranon fractures	2 (2.4)	3 (4.6)	0.6551^a^
Coronoid fractures	6 (7.3)	4 (6.1)	1.0000 ^a^
Lateral column humerus fractures	4 (4.9)	0 (0.0)	0.1299 ^a^
Scaphoid fracture	0 (0.0)	1 (1.5)	0.4422 ^a^
LCL	5 (6.1)	7 (10.8)	0.3699 ^a^

^a^ Fisher test

Sixty-one of the 65 subjects treated by mini-screws were clinically evaluated at a mean 47.3 ± 35.8 month follow-up, as it was not possible to contact 4 of the patients for the last clinical control. None of the patients treated using absorbable pins were absent at an average 82.5 ± 20.6 month of follow-up. The mean elbow ROM for the pin group was: flexion 138.2° ± 5.5, extension deficit 5.5° ± 11.7, pronation 73.1° ± 3.4, and supination 82.3° ± 6.0, whereas, the mean ROM for the patients treated using mini-screw was: flexion 139.5° ± 2.2, extension deficit 2.0° ± 7.0, pronation 73.6° ± 2.3, and supination 81.7° ± 5.4 (Table 2).

**Table 2:** Clinical results and statistical analysis

	PIN (mean ± SD) n = 82	Mini-screw (mean ± SD) *n* = 61	*p*-value	Post-hoc analysis of power^c^
Patient’s age (years)	45.4 ± 13.1	47.2 ± 15.4	0.459^a^	
Follow-up (months)	82.5 ± 20.6	47.3 ± 35.8	< 0.001^b^	
Flexion (°)	138.2 ± 5.5	139.5 ± 2.2	0.217^b^	58.2%
Extension deficit (°)	5.5 ± 11.7	2.0 ± 7.0	0.085^b^	49.7%
Pronation (°)	73.1 ± 3.4	73.6 ± 2.3	0.623^b^	18.7%
Supination (°)	82.3 ± 6.0	81.7 ± 5.4	0.290^b^	8%
DASH	0.8 ± 2.0	0.3 ± 0.5	0.554^b^	72.9%
MEPS	97.3 ± 5.8	98.3 ± 5.7	0.072^b^	17.8%
Comparison of displacement of the fragments			1.000^c^	
Comparison of residual pain			0.109^c^	

^a^ T-test

^b^ Mann-Whitney test

^c^ Fisher’s test

**Fig. 3:**
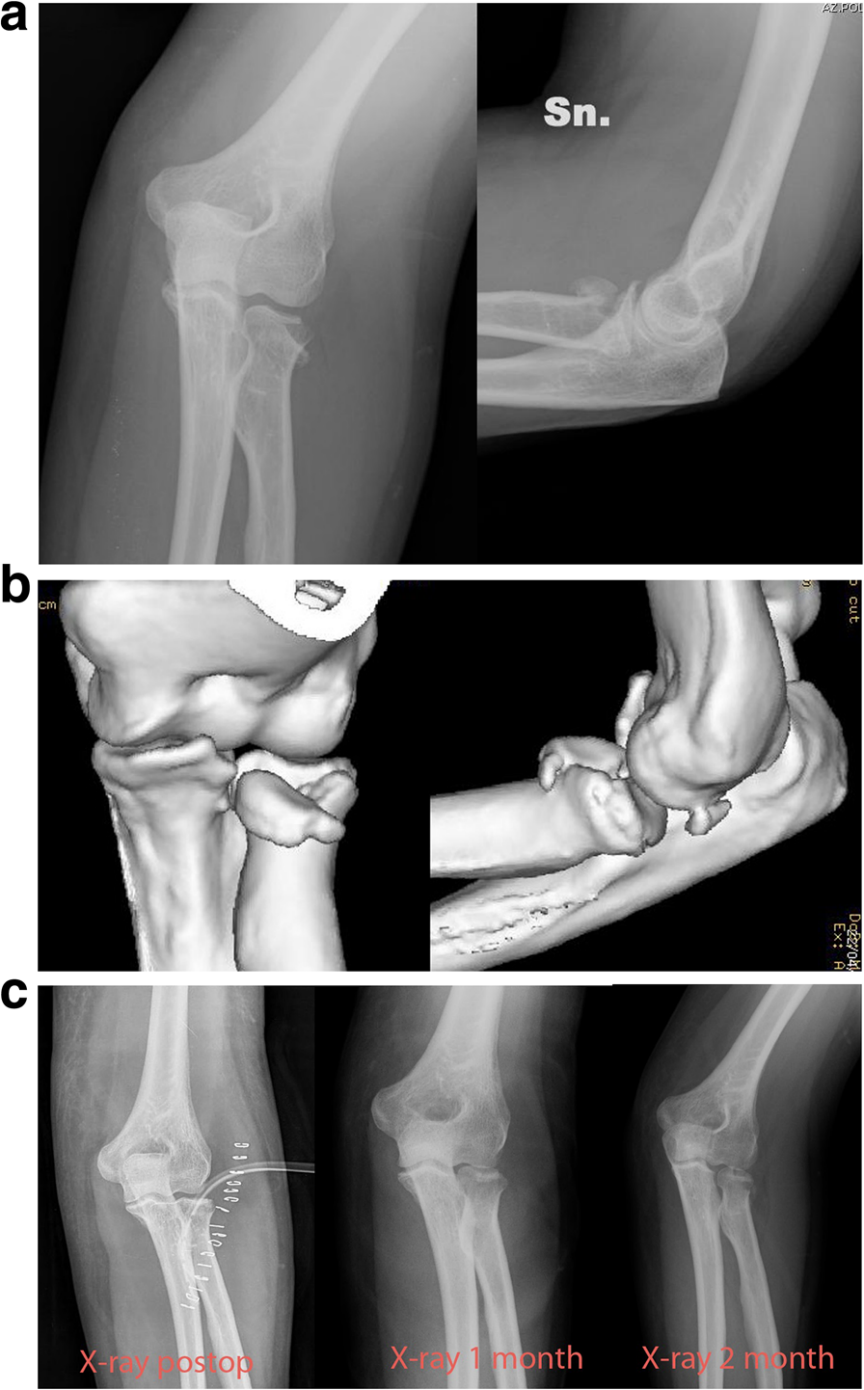
**a** Severely displaced Mason type II fracture. **b** 3D-computed tomography study. **c** X-rays follow-up with secondary displacement of fracture fragments of about 3 mm observed 2 months after surgery

**Fig. 4:**
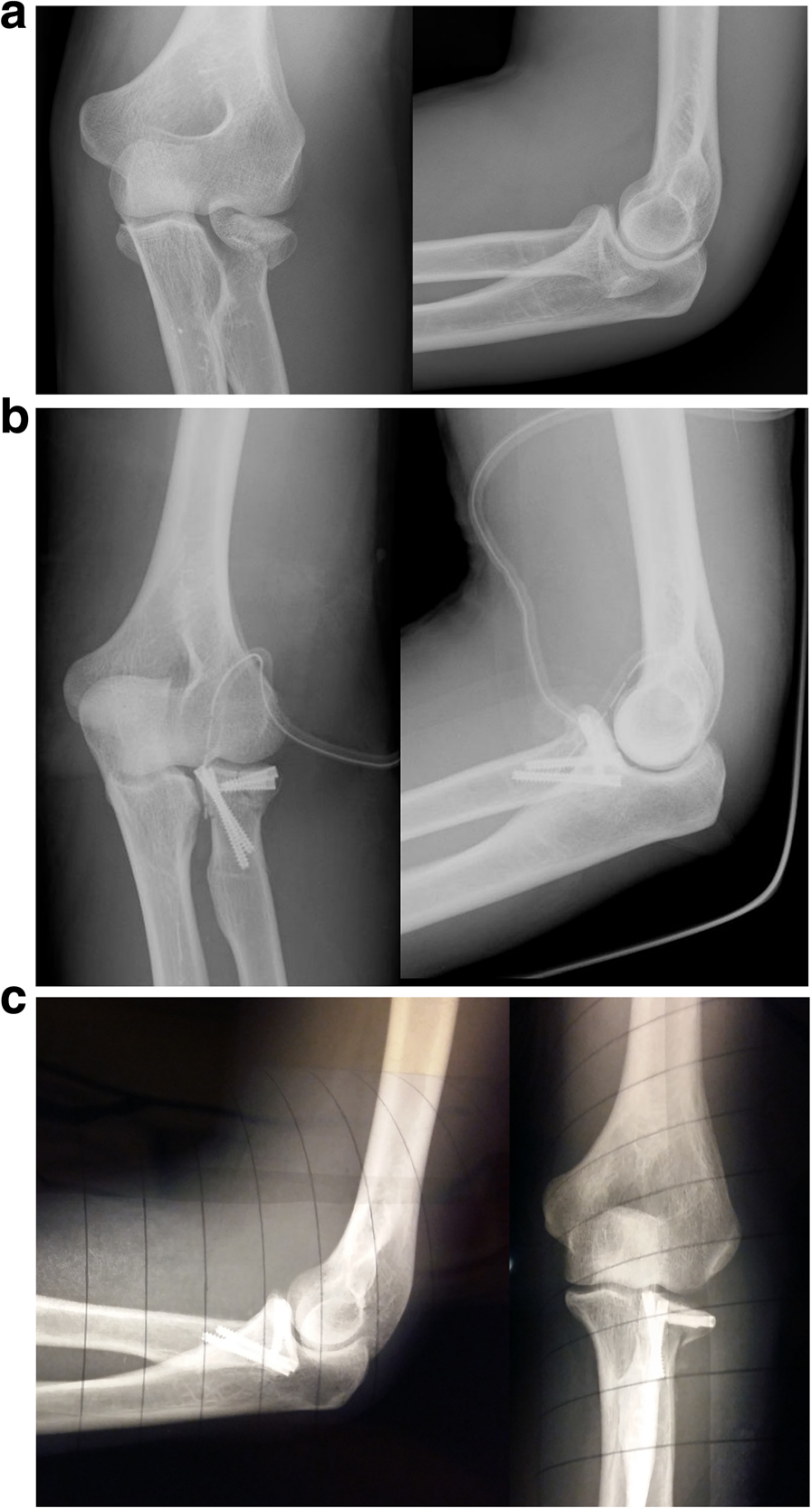
**a** X-rays showing a Mason type III fracture. **b** Post-operative x-rays with acceptable reduction using mini-screws. **c** X-rays showing secondary displacement of fracture fragments of about 4 mm observed 1 months after surgery

The mean DASH score was 0.8 ± 2.0 for patients treated using pins, and 0.3 ± 0.5 for the mini-screw group. Considering the MEPS, a mean score of 97.3 ± 5.8 was reported in the pin group, and 98.3 ± 5.7 for the patients treated using mini-screws. Comparing the ROM, DASH, and MEPS scores between the two groups no significant differences were observed (Table 2). No cases of symptoms of elbow instability, early or late occurrence of clinically evident seroma formation, osteolytic changes of the radial head, non-union, or infection was observed in either group. Residual pain was reported in 13 (15.8%) patients treated using pins, and in 6 (9.2%) patients treated using mini-screws. Radiological anatomical reduction was achieved intraoperatively in all cases; however, 7 (8.5%) patients treated using pins presented a secondary displacement of the fracture fragments of more than 1 mm (range 1–3 mm) in the postoperative radiographs (Fig. 3) vs. 1 case (1.6%) among the patients treated with mini-screws (Fig. 4). At the final clinical evaluation, no functional limitation which could influence social day to day life was highlighted in all subjects. Post-hoc power analysis is 8.2%; the minimum number of subjects for adequate study power is 3252 (1626 patients for each group – alpha error rate 0.05, beta error 0.2 and power of 80%). There are no significant differences for complications such as pain or fragments displacement (Table 2).

## Discussion

The properties of an ideal fixation for articular fractures include adequate strength and rigidity, lack of adverse side effects, any interference with bone healing and avoidance of an implant removal operation. Griffin et al. demonstrated that cross-cannulated 3 mm screws offered better rigidity than conventional T-plates in a cadaveric non-comminuted radial neck fracture model [12]. Koslowsky et al. performed a biomechanical study using radius saw bone models in Mason type III fractures comparing the quality of reduction, the failure load and the displacement of the reconstructed radial head at 50 N between fine threaded wires, T-mini-plates, 2 mm mini-screws and 2 mm K-wires [13]. The authors achieved a much better quality of reduction using fine threaded wires, followed by mini-screws and k-wires, and poor results with mini-plates. The ultimate failure load was similar for fine threaded wires, mini-screws and K-wires, but poor for mini-plates. The mean displacement at 50 N was significantly greater for plate fixation, than for fine threaded wires, mini-screws and k-wires [13]. Recently, absorbable pins have been proposed and tested in several studies [14-16, 20].

Hirvensalo et al., prospectively evaluated 24 patients treated with 2.0 mm absorbable polyglycolide pins for displaced radial head fracture, obtaining, at a mean 28 months of follow-up, excellent or good functional results in 22 patients (91%). Complications were represented by postoperative displacement of fractured fragments (range 1–3 mm) reported in 4 (16.7%) patients with severely comminuted fractures, and transient inflammatory reaction around the implants occurred in 2 (8.3%) cases 8–12 weeks postoperatively [17]. Pelto et al., reviewed, at an average 27 months of follow-up, 38 patients were treated with absorbable polyglycolide pins for Mason type II-III radial head fractures, reporting excellent or very positive functional results in 36 patients (95%), with 1 case of postoperative re-displacement and no adverse side effects from the implant [18].

Helling et al., published in 2006 a prospective, randomized, controlled multicentre study comparing standard mini-fragment metal implants (mini-screws, k-wires, mini-fragment plates) with biodegradable polylactide implants in 164 subjects with AO type B2.1, B2.2 and B2.2 radial head fractures [19]. At a mean 2-years follow-up, 96% of the polylactide patients and 92% of the control patients had excellent or good results, with an average Bromberg and Morrey Elbow Score of 93.3 in the polylactide group, and 90.9 in the control group (differences not statistically significant). Sixty-nine of the polylactide patients (84.1%) and 66 of the control patients (80.4%) were completely complication-free. Complications were represented by secondary fragment displacement in 5 cases in polylactide group and in 2 control patients, and osteolysis in 1 case for each group [19].

Givissis et al., retrospectively reviewed 21 patients with Mason type II-III-IV fractures treated with absorbable pins, reporting at a mean 81 months of follow-up (range, 36–136 months). The study reported good fracture healing with no radiographic signs of osteolysis in every case, no material-related adverse reactions, mean MEPS score of 93.8 (range, 20–100), and mean elbow ROM of 9°-132° in flexion, 79° pronation, and 77° supination [20]. These results are comparable in terms of functional score and ROM with those obtained in the present research using absorbable pins and mini-screws (Table 2).

The authors reported the same satisfactory clinical results for both methods: the means MEPS score is 97.3 +/− 5.8 in pin population and 98.3 +/− 5.7 in screws population, the mean DASH score is respectively 0.8 +/− 2 and 0.3 +/− 0.5. Different studies recorded higher DASH score [21], we believe that such poor results could be down to different pattern of fractures. The trend of better results in terms of outcomes for patients treated by mini-screws is not statistically significant. The minimal limitation of excursion and moderate pain, that had been registered did not influence day to day life and each patient expressed great satisfaction for their surgical treatment (Table 2). It is of importance to note that the residual pain and limitation of excursion, are not related to secondary displacement. This factor, could possibly be linked to a different post-operative protocol of immobilization that could lead to stiffness. However, we preferred to immobilize the patients who we had treated with pins, according to literature [17, 18] because of minor axial strength [19]. The lack of detailed clinical data regarding functional outcomes of two groups at 30 and 60 days after surgery limited our thoughts regarding the range of motion recovery.

### Limitation

A possible limitation of this study is represented by the different follow-up duration of the 2 groups, respectively 82.5 ± 20.6 for the pin group vs. 47.3 ± 35.8 for the mini-screw group. This is due to replacement of the surgical practice using pins in the last five years in our institute. This technical decision was based on the surgeon’s personal preference. The collection of data, over a long period of time, is a significant area of potential study bias as the surgeon could have possibly experienced substantial technical improvement. Furthermore, the measurements regardless of the final results of the clinical check up carried out by one of the authors, could also affect our study. It is important to note that the post-operative protocol has not changed and the classification of fracture pattern, was carried out by the same author.

Unfortunately, it was impossible for the author to find any clinical data, regarding functional results between the two groups at 30 and 60 days in our records. The possible late recovery of range of motion could be an important factor influencing the choice of treatment.

Another limitation is the absence of a long-term radiological evaluation to assess possible delayed adverse effects of the bio-absorbable material used for pins and the onset of radio-humeral arthrosis.

A further limitation of the study is the low statistical power. Although Post-Hoc Analysis was discussed as a post-hoc method, analysis would require a larger sample of patients to achieve results with greater power. Finally, post-hoc analysis of power is poor possibly due to the restricted sample size. For any further studies, it may be helpful to increase the sample size to avoid the risk of type II error that could affect our work and results.

## Conclusions

Both absorbable pins and mini-screws provide good clinical and functional scores at a mid-term follow-up. However, a higher rate (8.5% of cases) of secondary displacement of the fracture fragments was reported among those subjects treated using absorbable pins.

On the other hand, little incongruence of the articular surface could not determine major complications in term of function and pain after an average follow-up of 10 years, as we have shown in our study. We suggest adopting a post-operative protocol including longer immobilization, perhaps a further twenty days, in a cast or elbow brace and at the same time to start physiotherapy treatment taking into consideration cautious mobilization to avoid any stiffness.

## Abbreviations

DASH: Disabilities of the arm, shoulder and hand
LCL: Lateral collateral ligament
MEPS: Mayo elbow performance score
ORIF: Open-reduction and internal fixation
ROM: Range of motion
